# Long-Term Non-Progression and Broad HIV-1-Specific Proliferative T-Cell Responses

**DOI:** 10.3389/fimmu.2013.00058

**Published:** 2013-03-01

**Authors:** Nesrina Imami, Samantha J. Westrop, Nathali Grageda, Anna A. Herasimtschuk

**Affiliations:** ^1^Department of Medicine, Imperial College LondonLondon, UK

**Keywords:** HIV-1, T lymphocytes, cell proliferation, disease progression, LTNP

## Abstract

Complex mechanisms underlying the maintenance of fully functional, proliferative, HIV-1-specific T-cell responses involve processes from early T-cell development through to the final stages of T-cell differentiation and antigen recognition. Virus-specific proliferative CD4 and CD8 T-cell responses, important for the control of infection, are observed in some HIV-1^+^ patients during early stages of disease, and are maintained in long-term non-progressing subjects. In the vast majority of HIV-1^+^ patients, full immune functionality is lost when proliferative HIV-1-specific T-cell responses undergo a variable progressive decline throughout the course of chronic infection. This appears irreparable despite administration of potent combination antiretroviral therapy, which to date is non-curative, necessitating life-long administration and the development of effective, novel, therapeutic interventions. While a sterilizing cure, involving clearance of virus from the host, remains a primary aim, a “functional cure” may be a more feasible goal with considerable impact on worldwide HIV-1 infection. Such an approach would enable long-term co-existence of host and virus in the absence of toxic and costly drugs. Effective immune homeostasis coupled with a balanced response appropriately targeting conserved viral antigens, in a manner that avoids hyperactivation and exhaustion, may prove to be the strongest correlate of durable viral control. This review describes novel concepts underlying full immune functionality in the context of HIV-1 infection, which may be utilized in future strategies designed to improve upon existing therapy. The aim will be to induce long-term non-progressor or elite controller status in every infected host, through immune-mediated control of viremia and reduction of viral reservoirs, leading to lower HIV-1 transmission rates.

## Introduction

HIV-1 has infected more than 65 million people worldwide and this number is increasing year on year, with 34 million persons currently living with the virus (UNAIDS, 2012). The virus/host relationship is not sustainable, and, without chemotherapy, 99% of infected-individuals succumb to AIDS, resulting in death within an average of 10 years. SIV, the simian ancestor of HIV, has been present in its natural host for centuries and establishes a non-pathogenic, life-long infection. In contrast, HIV-1 infection, a relatively recent emergence, is almost universally fatal to its host. Huge global efforts are being implemented to provide efficacious protection from infection for future generations through HIV prevention, treatment, and the development of prophylactic vaccines^1^^,^^2^. Many millions of pounds have been invested in the development of non-curative combination antiretroviral chemotherapy (cART), an indisputable success for the treatment of HIV-1-infected individuals. Our understanding of the pathogenic interactions between a number of hosts and different retroviruses continually increases. Short, more user-friendly treatment regimens, with persisting therapeutic benefits, are required to attenuate disease progression and enable host and pathogen to co-exist; rather than life-long, costly, and often unpleasant, treatment. It is noteworthy that the number of deaths from AIDS worldwide and in the UK, although initially reduced following the introduction of cART, now shows little evidence of major decline according to the most recent report from Health Protection Agency (HPA, 2012). Employing the vast scientific knowledge of the immune system in humans, and our evolutionary neighbors the non-human primates, understanding immune control of viral infections and translating that knowledge into the development of effective immunotherapies, while challenging is of substantial importance. Although viral eradication from the infected host must remain the ultimate goal, a more feasible therapeutic target may be the co-existence of host and virus in the absence of therapeutic drugs, as observed in long-term non-progressing individuals or elite controllers who represent <1% of the HIV-1-infected cohort and control plasma HIV-1 RNA levels to below limit of detection (BLD) (Blankson, 2010; Migueles and Connors, 2010; Mandalia et al., 2012). The discovery of a successful prophylactic immunization strategy will still leave approximately 34 million HIV-1^+^ people worldwide in need of improved, long-term therapeutic options. The information available is vast, therefore we have summarized some of the findings that address key questions on how individuals with HIV-1 non-progressive infection, characterized primarily by proliferative and IL-2-producing HIV-1-specific CD4 and CD8 T-cell responses, are able to keep the virus under control.

## The Long-Term Non-Progressor: Criteria of Definition and Emulation of LTNP Status

Cohorts of long-term non-progressors (LTNP) were identified prior to the availability of assays that quantify the plasma viral load (pVL), and as a result viremia was not initially included in the criteria of definition (Pantaleo and Fauci, 1996). Following the introduction of the viral load assay, the term “True LTNP” defined individuals meeting the original LTNP criteria in addition to suppressed pVL BLD (Migueles and Connors, 2010). Terminology then changed to refer to such atypical HIV-1^+^ individuals as “Elite controllers,” “Elite suppressors,” and “HIV controllers” (Deeks and Walker, 2007; Blankson, 2010; Migueles and Connors, 2010). More recently, an additional group of individuals exhibiting a disparate course of clinical disease have been described and termed “Discord controllers” (Groves et al., 2012). These individuals control viral replication to BLD of the routinely available viral load assay, but demonstrate peripheral blood CD4 T-cell counts lower than the normal range, therefore failing to meet the inclusion criteria for LTNP status. It is thought that definitions based upon the “normal range” of CD4 T-cell counts can be somewhat stymied as they fail to take into account natural variations in CD4 T-cell count within and between populations of different ethnicities, and that the infecting viral clade may differ from that which current routinely available pVL assays have been optimized to detect (Westrop et al., 2011).

In our cohort, HIV-1^+^ patients exhibiting stable CD4 T-cell counts below normal range displayed shorter time to disease progression than LTNP who maintained their CD4 T-cell counts within the normal range (Mandalia et al., 2012). However eventual disease progression appeared very likely in both groups – indicating loss of control over HIV-1. The combined data so far show that only a few LTNP and elite controllers have been identified in any cohorts, endorsing the need for universal definitions to facilitate more meaningful comparisons (Mandalia et al., 2012). The major question remains as to why disease progression eventually occurs in our cohort of LTNP after many years of effective viral control (Westrop et al., 2009b; Mandalia et al., 2012), and studies to date suggest multiple factors.

Data presented in various studies accentuate a need for collaborative efforts when studying these atypical patients, such as work performed within the international Genetic and Immunological Studies on HIV^+^ European and African LTNP (GISHEAL) and the HIV Controllers Study (HIVCS) consortia (Pereyra et al., 2010; Guergnon et al., 2012), to increase the number of LTNP and controllers who can be identified and studied. Using different criteria to define patient groups results in different estimates in time until disease progression, before even considering functional immunology, genetic, and virological factors, further emphasizing the need for agreed standardized use of terminology and definitions before more in depth study of these individuals is performed. LTNP within the Chelsea and Westminster HIV cohort are defined by a duration of HIV-1 infection longer than 7 years from time of HIV-1^+^ diagnosis, in the absence of cART and clinical symptoms, and the stable maintenance of CD4^+^ T-cell count within the normal reference range (Mandalia et al., 2012). For this review, we use the term LTNP to refer to HIV-1^+^ subjects that fit the criteria used to define our cohort, and with pVL BLD.

It has been well established for over two decades that HIV-1 infection is branded with loss of T-cell proliferation accompanied with limited IL-2 production (Lane et al., 1985; Shearer et al., 1986; Miedema et al., 1988; Clerici et al., 1989; Gruters et al., 1990; Fan et al., 1993). Importantly, “True LTNP” show proliferative responses associated with cytotoxic potential of HIV-1-specific CD8 T cells, known to correlate with immunologic control of HIV-1 replication (Migueles et al., 2002, 2008). Such elite suppression (Blankson and Siliciano, 2008), trademarked by effective proliferation and clonal expansion, is linked with higher levels of cytolytic granules (granzyme B and perforin) within HIV-1-specific CD8 T cells. This is observed only in these unique LTNP who control HIV-1 replication, but not in typical progressors. Several different mechanisms of viral control have been proposed, nonetheless findings clearly suggest that proliferation is a prerequisite for producing effective CD8 T cells able to kill target CD4^+^ cells (Blankson and Siliciano, 2008; Migueles et al., 2008). Furthermore, these findings by Connors and colleagues (Migueles et al., 2008) also showed that stimulation of cells from typical progressors and induction of proliferation resulted in effective killing – indicating the potential to reverse CD8 T-cell unresponsiveness in progressors.

Over the last few years several reports, including our own, have supported the idea that in LTNP with undetectable viral load, pivotal CD4 helper T lymphocyte (HTL) orchestration of anti-HIV-1 immunity is mediated via intricately balanced proliferative ability and cytokine profiles (Wilson et al., 2000; Imami et al., 2002). Our current understanding of the response to SIV and HIV-2 infection in the natural hosts, and the fact that amongst the broad spectrum of HIV-1 pathological manifestations (Klein and Miedema, 1995), a very small number of HIV-1-infected persons (<1%) remain well without suffering quantitative or qualitative declines in proliferative IL-2-secreting HIV-1-specific CD8 and CD4 T-cell responses (Wilson et al., 2000; Imami et al., 2002; Migueles et al., 2002, 2008; Betts et al., 2006; Westrop et al., 2009b), indicates that modulation of the immune system may offer benefits to chronically infected individuals receiving cART. Individuals who successfully suppress viral replication to BLD retain the immune profile present in most patients at the early stage of infection; that is, they display proliferative virus-specific T-cell responses, fully functional antigen-presenting cells (APC) including higher numbers of pDC, and intact innate immune responses, for many years following infection (Borrow et al., 1994; Koup et al., 1994; Rinaldo et al., 1995; Harrer et al., 1996; Rosenberg et al., 1997; Gea-Banacloche et al., 2000; Wilson et al., 2000; Soumelis et al., 2001; Imami et al., 2002; Migueles et al., 2002).

## Interplay Between Viral Pathogenesis and Immunopathology

Despite over 30 years of concentrated study, many aspects of HIV-1 immunopathology remain unclear. A very important model of the pattern of natural pathogenic infection has emerged, based on the SIV-infected rhesus macaque, which describes the immunopathogenesis of lentiviral infection as the result of two distinct periods of destruction (Li et al., 2005; Mattapallil et al., 2005). The two-phase infection is characterized by an acute, highly destructive phase when virus massively depletes the CD4 memory T cells from effector sites in the gastrointestinal tract, followed by a chronic phase in which the damaged immune system slowly fails as constant immune hyperactivation eventually exhausts the ability for reconstitution (Li et al., 2005; Mattapallil et al., 2005; Douek et al., 2009). This early destruction of the CD4 memory T-cell pool coupled with rapid establishment of a viral reservoir suggests that current strategies alone, which work to suppress replication of virus, are unlikely to result in eradication of the viral reservoir from the host, or facilitate reconstitution of immunity (Richman et al., 2009; Deeks et al., 2012). Nonetheless, in LTNP and other HIV-1^+^ individuals receiving multi-targeted drug treatment at the initial acute stages of infection, the proviral reservoir has been described as significantly reduced compared to those treated at a later chronic stage of infection (Pires et al., 2004; Cellerai et al., 2011; Ananworanich et al., 2012).

Strong virus-specific CD8 cytotoxic T lymphocyte (CTL) responses are required to control most viral infections including HIV-1 (Walker et al., 1987; Koup et al., 1989, 1994; Klein et al., 1995; Moss et al., 1995; Goonetilleke et al., 2009). CD4 HTL also play a principal role in supporting the development of the humoral immune response, during antigen processing and presentation by APC, and have a major influence on the development, maturation, differentiation, and maintenance of effective CD8 CTL immunity (Kaech and Ahmed, 2003; Lichterfeld et al., 2004; Williams and Bevan, 2007). The complex interactions between the virus and T cells involve recognition of antigen by T cells, a process that requires antigen capture and processing into peptides (York and Rock, 1996; Rudolph et al., 2006; Blum et al., 2013). Such peptides originating from the cytosol within an infected cell, are delivered and loaded to form tertiary structure complexes with Major Histocompatibility Complex (MHC) class I molecules, and are displayed on the cell surface for recognition by the CD8 T-cell receptor (TCR). Peptides derived from vesicles are recognized by CD4 TCR, whilst in complex with MHC class II molecules on the surface of an APC (Sebzda et al., 1999; Blum et al., 2013). These interactions initiate the anti-viral immune response, including proliferation, clonal expansion, and differentiation (Lanzavecchia and Sallusto, 2002; Sallusto and Lanzavecchia, 2011). Generation of CD8 CTL memory is dependent on the presence of fully functional CD4 HTL during both priming and effector function (Zajac et al., 1998; Janssen et al., 2003; Shedlock and Shen, 2003; Sun and Bevan, 2003). Douek et al. (2002) have shown that peripheral HIV-1-specific CD4 HTL are preferentially targeted for viral infection, and that these cells are possibly anergized during acute infection, and subsequently deleted or destroyed over the course of chronic disease. In SIV infection of sooty mangabeys or African green monkeys no immunosuppression is evident, and in HIV-2 infection progression to disease is very slow, even though viral loads may be high. Mechanisms for the cessation of, or reduced rates of, disease progression in SIV or HIV-2 infection remain undefined, although it has been suggested that distinct T-cell activation and cytokine profiles reflect the presence of effective host immune responses and the extent of viral replication (Hirsch, 2004; Hanson et al., 2005; Silvestri, 2005). The essential role of T-cell proliferation coupled with IL-2-producing capacity was identified in HIV-2 studies describing maintenance of virus-specific CD4 T-cell help, and polyfunctionality of both CD4 and CD8 T cells as hallmarks of non-progressive infection (Alatrakchi et al., 2006; Duvall et al., 2006, 2008), providing additional support for the role of proliferative IL-2-producing T cells in viral control. In HIV-1^+^ LTNP, unlike the chronically infected typical progressors, the majority of T cells are not rendered anergic either by HIV-1 or by clonal inactivation, exhaustion, or suppression. HIV-1-specific CD8 T cells are not deficient in their differentiation, maturation, and proliferative function, and the virus does not escape through generation of mutations in viral epitopes targeted by T cells (Allen et al., 2000; Kelleher et al., 2001; Moore et al., 2002; Seder and Ahmed, 2003; Bailey et al., 2006). Strong proliferative HIV-1-specific CD4 T-cell responses to a number of viral proteins are demonstrable (Rosenberg et al., 1997; Wilson et al., 2000; Imami et al., 2002; Iyasere et al., 2003; Malnati et al., 2012), and APC and natural killer (NK) cell function are normal (Mendila et al., 1999; Stebbing et al., 2003). The presence of a fully functional anti-HIV-1 immune response in LTNP coupled with undetectable or very low pVL, consequently results in low transmission potential (Migueles et al., 2000). In contrast to LTNP, the majority of HIV-1^+^ patients are chronically infected, have progressive disease, become severely immunosuppressed (Pantaleo and Fauci, 1996), and if untreated remain highly infectious – fueling the AIDS pandemic.

Taking into account the kinetics of the immune response and steps in antigenic stimulation over time observed in LTNP, and applying that to immunotherapeutic and prophylactic settings presents a challenge for the future. Accumulation, availability, and presentation of antigen through cell-to-cell interactions and cytokine networks determine progression of T cells through stages of differentiation and proliferation (Lanzavecchia and Sallusto, 2002). Following antigenic stimulation, IL-2 production precedes T-cell proliferation providing there is effective IL-2/IL-2R interaction, signaling, and responsiveness (Smith, 2001; Malek, 2008). This promotes the development and homeostatic maintenance of T-cell memory (through cell-division/proliferation), and triggers functional down-regulatory or inhibitory cascades essential for a balanced proliferative HIV-1-specific T-cell response observed in LTNP (Imami et al., 2002; Downey and Imami, 2010). It is through such mechanisms that LTNP appear to sustain survival of IL-2 responsive (and producing) T cells that are readily able to mount a proliferative response.

The scientific rationale for the use of immunotherapy in juxtaposition with cART as novel therapeutic intervention is based on the demonstrable increase in naïve T-cell numbers upon commencement of effective cART, when viremia is fully suppressed (Kelleher et al., 1996; Autran et al., 1997; Connors et al., 1997). This is thought to indicate residual thymic function producing new T lymphocytes (Haynes et al., 2000; Serana et al., 2011; Quiros-Roldan et al., 2012), and IL-7-driven homeostatic survival and proliferation of naïve T cells (Takada and Jameson, 2009; Crawley and Angel, 2012). This supports the feasibility of therapeutically induced immune reconstitution in HIV-1^+^ patients. We and others have demonstrated that recovery of immune responsiveness during administration of cART is reflected in functional improvement of T-cell responses directed toward various recall antigens and other pathogens (Autran et al., 1997; Li et al., 1998; Hardy et al., 2003). However reconstitution of fully functional, proliferative, HIV-1-specific CD8 T-cell (Appay et al., 2000; Gea-Banacloche et al., 2000; Migueles et al., 2002), and HIV-1-specific CD4 T-cell responses (Kelleher et al., 1996; Autran et al., 1997; Connors et al., 1997; Wilson et al., 2000; Iyasere et al., 2003; Younes et al., 2003) remains incomplete. It is also apparent that reduced numbers of APC, together with loss of APC function, contributes to HIV-1-specific T-cell anergy, including the suppression of proliferative T-cell responses (Knight and Patterson, 1997; Chougnet et al., 2002; Boasso et al., 2007, 2008; Sabado et al., 2010), and that NK cell function is deregulated by HIV-1 infection (Mela et al., 2005). Such dysfunctional immune responses are apparent very early after HIV-1 infection, and may be favorably affected by early initiation of multi-targeted ART and long-term duration of successfully suppressive ART (Ananworanich et al., 2012). It remains clear that induction and maintenance of HIV-1-specific T-cell responses in chronically infected individuals with progressive infection requires the generation and preservation of proliferative effector and helper T-cell subsets and innate immune responses similar to those seen in LTNP.

## CD4 T Cells in HIV-1 Disease and Control and Their Proliferative Aptitude

Long-term non-progressor individuals mount vigorous and broad IL-2-producing CD4 HTL proliferative responses to multiple HIV-1 antigens, particularly to the core proteins (Rosenberg et al., 1997; Imami et al., 2002). Using conventional and novel immunologic assays in parallel, we have demonstrated that functional defects in HIV-1-specific CD4 HTL in chronic HIV-1 infection include an inability to proliferate and produce IL-2 in response to HIV-1 antigens, although secretion of anti-viral cytokines such as IFN-γ and TNF-α remained unimpaired (Wilson et al., 2000). These important findings demonstrated that HIV-1-specific CD4 T cells might not be irretrievably deleted during chronic infection, but are actually present although unable to respond adequately to HIV-1. Subsequent studies concurred with these initial findings, and detailed simultaneous analysis of IFN-γ and IL-2 production by anti-HIV-1 CD4 T cells, and their resulting proliferation in clinical progressors and LTNP revealed three functionally distinct subsets of virus-specific CD4 T cells: those producing IFN-γ only; those producing IL-2 only; and cells producing both IFN-γ and IL-2 (Iyasere et al., 2003; Younes et al., 2003; Harari et al., 2004a). Further phenotypic analysis of CCR7/CD45RA expression (Sallusto et al., 1999), revealed a phenotypic heterogeneity of virus-specific CD4 T cells, dictated by both viral load and persistence (Harari et al., 2004b); highlighting the need to recognize the relative functional and phenotypic heterogeneity of T-cell subsets specific to HIV-1 and other viruses (Appay et al., 2008), particularly in the context of co-infection. This heterogeneity may be due to different types of antigen and APC employed, resulting in altered proliferative capacity and a variable CD4 HTL response.

When considering the potential of therapeutic immunization and other forms of immunotherapy for HIV-1^+^ patients, it is important to consider the incongruent reconstitution of the memory and naïve CD4 T-cell compartments upon treatment with cART (Schacker et al., 2006). Long-term use of cART during chronic HIV-1 infection may increase numbers of memory and naïve CD4 T cells specific for opportunistic pathogens, but does not apparently allow regeneration of proliferative HIV-1-specific CD4 T-cell responses with the potential to keep the virus under control (Autran et al., 1997). In the vast majority of cases, the exception perhaps being when cART is administered extremely early after initial infection (Rosenberg et al., 1997, 2000), cART does not allow LTNP status to be established. Thus such treatment does not promote reconstitution or regeneration of proliferative HIV-1-specific CD4 T-cell responses with the potential to control viremia and protect CD4^+^ cells from infection and destruction – facilitating discontinuation of cART. It has been considered that the substantial damage occurring early in disease is irreparable (Brenchley et al., 2006). However we suggest that rather than being deleted, certain HIV-1-specific CD4 T-cell clones are present but anergized, and consequently fail to provide critical help to anti-HIV-1 CD8 CTL. The presence of HIV-1-specific CD4 HTL, albeit not fully functional, implies a potentially reversible process to allow the kinds of proliferative responses observed in LTNP (Imami et al., 1999; Wilson et al., 2000). We reported robust and broad virus-specific proliferative CD4 HTL responses in LTNP to a range of HIV-1 proteins and peptides such Gag and Env (Wilson et al., 2000; Imami et al., 2002), and additionally responses to regulatory Nef, Tat, and Rev (Westrop et al., 2009b; Malnati et al., 2012). It is also important to consider the CD4 T-cell functionality in gastrointestinal tract, since it has been reported that controllers have higher magnitude and frequency of polyfunctional mucosal HIV-1-specific CD4 T cells for which the strongest responses were associated with certain MHC class II alleles (HLA-DRB1*13, HLA-DQB1*06) (Ferre et al., 2010).

Long-term non-progressors with controlled HIV-1 replication also mount significantly higher proliferative responses to other viruses, namely influenza, HSV, VZV, and CMV compared to untreated chronically infected HIV-1^+^ patients (Imami et al., 2001). This more robust lymphoproliferative response is also seen when LTNP are compared to HIV-1-negative individuals where anti-HSV and -CMV proliferation in LTNP exceeds that of healthy controls (Imami et al., 2001). A possible explanation for this may be the difference in prevalence of HSV and CMV infection between the two cohorts. Alternatively this data may demonstrate a superior anti-viral proliferative ability in LTNP, which is not limited to the anti-HIV-1 response. Responses to and control over other viruses such as HCV and HBV are comparable to those observed within HIV-1-negative individual (Lauer et al., 2002; Thomas, 2008). Distinctive divergent T-cell proliferative response observed between HIV-1 and other viruses might be due to relative differences in viral loads and/or viral replication (Day and Walker, 2003), which might or might not result from viral fitness/evasion of host immunity (in both HIV-1 and HCV) (Grakoui et al., 2003). HCV viral load has been reported lower in HIV-1 controllers coinfected with HCV, than in chronically HIV-1-infected counterparts (Ruiz-Mateos et al., 2011). Similarly to HIV-1 infection, this study also associated HLA-B*57 with superior control of HCV, and HLA-B*35 with worse control, however there were no HLA-associated differences in spontaneous HCV clearance rates (Ruiz-Mateos et al., 2011), suggesting common host mechanisms involved in the control of plasma viremia in these two persistent viral infections.

An additional possible mechanism in individuals responding not only to HIV-1 but also other viruses, could relate to the virus-specific CD8 T-cell evasion of regulatory CD4 (or CD8) T-cell (Treg) suppression (Elahi et al., 2011). CD8 CTL specific for HIV-1, HSV-2, and EBV HLA-B*27 and HLA-B*57 restricted epitopes were resistant to Treg cell-mediated suppression, explaining how such cells continue to proliferate and control infection(s) in LTNP, which might also apply to the virus-specific CD4 T cells.

Furthermore, CD4 T cells from “Elite controllers” have been shown to resist HIV-1 infection, reverse-transcribing viral genomes, and transcribing mRNA from proviral DNA less effectively than CD4 T cells from chronically infected HIV-1^+^ individuals (Chen et al., 2011). This resistance has been associated with upregulation of intracellular p21, a cyclin-dependent kinase inhibitor (Chen et al., 2011), and may explain the lower proviral DNA reservoirs in peripheral blood and in the central memory CD4 T cells of LTNP (Pires et al., 2004; Descours et al., 2012). Lower levels of HIV-1 provirus may result in reduced expression of viral proteins by these CD4 T cells, and therefore a lower density of viral epitopes presented on the cell surface to surveying HIV-1-specific CD8 CTL, resulting in fewer CD4 T cells targeted by CTL killing (Descours et al., 2012). Therefore another pertinent question to be answered is how the HIV-1-infected target CD4^+^ cells protect themselves from CTL attack? There are a number of potential mechanisms accounting for this, as already hinted at, others might include upregulation of inhibitory immunoregulatory molecules such as CTLA-4 (Kaufmann et al., 2007), PD-1 (Day et al., 2006), and TIM-3 (Jones et al., 2008; Downey and Imami, 2010). Cell surface density of immunoregulatory molecules and their ligands on target cells and/or effector T cells may orchestrate cytotoxic killing, with enhanced expression of inhibitory markers overriding such activity, resulting in limited cytotoxic capacity.

It has been known for almost two decades that cellular immunity is involved in viral control during acute infection (Koup et al., 1994) and in long-term asymptomatics (Klein et al., 1995). However it is essential to compare methodologies utilized previously to those subsequently introduced, as well as the antigenic stimuli used (whole antigen/protein, peptides or APC employed), in order to comfortably distinguish between the precise contribution of virus-specific CD4 and CD8 T-cell responses. A recent report from Soghoian et al. (2012) adds an interesting twist – defining HIV-1-specific cytolytic CD4 T cells and placing them both at the center and to the forefront of the immune response. It was reported that in primary HIV-1 infection, controllers had an early expansion of both classical HTL, and cytolytic CD4 T cells which were able to kill infected cells directly. Such cytolytic CD4 T-cell responses have been shown to kill Gag peptide-pulsed autologous targets *ex vivo* (Norris et al., 2004). Moreover, these recent findings indicate that during acute infection such killer HIV-1-specific CD4 T cells are predictive of favorable outcome and are characterized by the expression of the death protein granzyme A (Soghoian and Streeck, 2010; Soghoian et al., 2012), implying that expansion of these cells during initial stages of HIV-1 infection controls viral replication.

Regulatory T cells (Tregs) in LTNP express more inhibitory Tim-3 receptor than chronic progressors and are therefore less active and do not suppress HIV-1-specific or other virus-specific CD8 T-cell responses (Elahi et al., 2011). It seems likely that virus-specific CD4 T cells of LTNP are able to evade Treg suppression. Although the impact of Tregs on proliferative virus-specific CD4 T-cell responses in LTNP and progressors remains controversial (Blankson, 2010; Burton et al., 2011), the first documentation of HIV-1-Gag-specific Tregs using MHC class II tetramer technology will shed light on the role of these cells in HIV-1 immunopathogenesis (Angin et al., 2012).

Importantly, the proportion of naive and memory effector, CD45RO^+^ T cells in LTNP are comparable to uninfected controls. The majority of CD4 T cells express the co-stimulatory molecule CD28 and do not express HLA-DR, suggesting a resting memory phenotype with full co-stimulatory ability most likely representing central memory T cells (T_CM_) (Boaz et al., 2002; Imami et al., 2002). Subsequent reports have indicated that LTNP, like patients initiating cART early, have high proportions of T_CM_. T cells that are low in HLA-DR and high in CD28 surface expression are most likely the same subset, because they rapidly proliferate and produce vast amounts of IL-2 *in vitro* in response to both HIV-1 and other viral antigens (Day and Walker, 2003; Younes et al., 2003; Harari et al., 2004a). Sustained CD28 expression also increases total proliferative potential (Parish et al., 2010). However, it is essential to understand not only surface markers of activation and exhaustion (Downey and Imami, 2010), but also the intracellular signaling events which are essential for priming/activation and maturation/differentiation of both CD4 and CD8 T-cell subsets. This may reveal specific differences in the signaling cascades which favor fully functional proliferative dynamics in non-progressors, and lead us to understand how unresponsiveness/anergy can be reversed (Downey et al., 2011).

## HIV-1 Plasma RNA Load, Viral Reservoirs, and Proliferation

**Figure 1:**
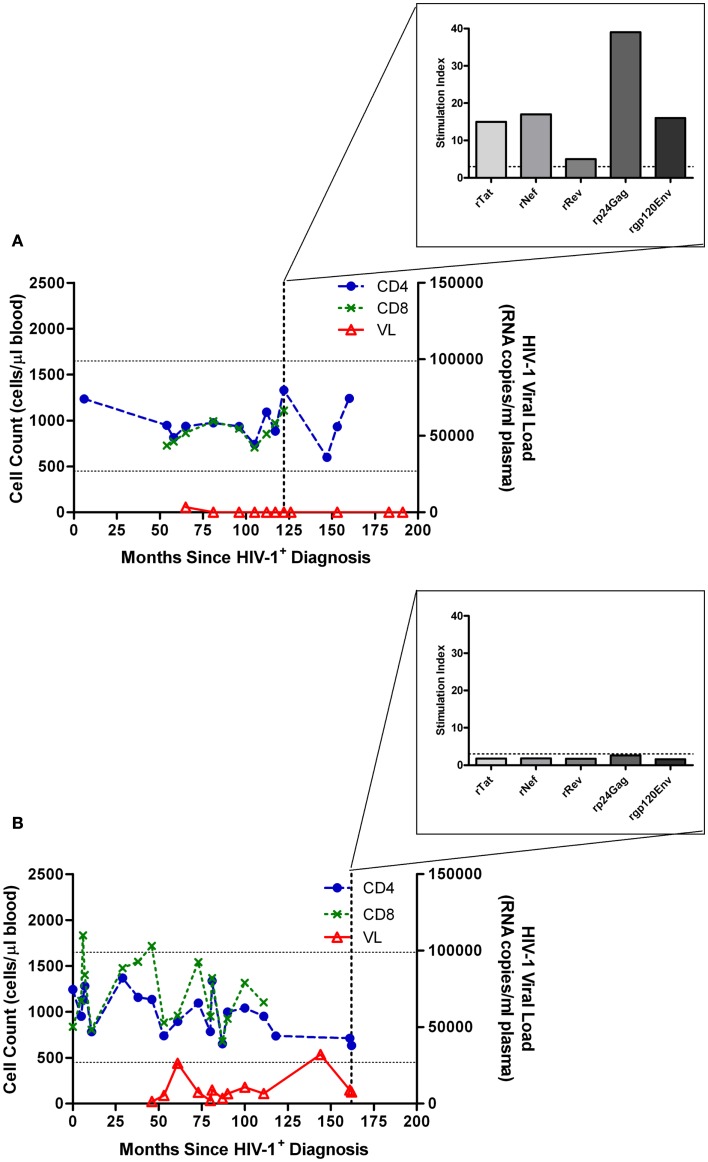
**Characteristic proliferative responses detected by 3H-thymidine incorporation in a lymphocyte proliferation assay**. **(A)** Illustration of the characteristic presence of proliferative responses to recombinant (r) Tat, rRev, rNef, rp24 (Gag), and rgp120 (Env) HIV-1 proteins observed in a “True LTNP” with suppressed HIV-1 plasma load, and **(B)** a typical lack of such responses in a chronically infected patient (Westrop et al., 2009b). Normal CD4 T-cell range of 450–1650 cells/μl blood is marked with horizontal lines. The threshold for positivity, stimulation index ≥ 3, is also marked with a dashed horizontal line (inset graph).

We have previously reported that even a small increase in pVL may be indicative of forthcoming clinically relevant changes of disease state. An increase from 1,236 to 6,483 RNA copies/ml plasma led to a loss of HIV-1 Gag-specific proliferative responses, and a shift toward a type II cytokine profile as indicated by loss of IL-2 production and increase in IL-4 production, and subsequent disease progression (Imami et al., 2002). This increase in pVL and loss of CD4 proliferation has also been described to occur in elite controllers who subsequently progress (Dyer et al., 2008). Comparison between two HIV-1^+^ individuals both presenting with atypical HIV-1 disease progression and non-declining CD4 counts is shown in Figure 1. Substantial proliferation to a number of HIV-1 proteins is demonstrated in a non-progressing individual with suppressed pVL (Figure 1A). Lack of such a proliferative response is seen when viremia is above detection limit (Figure 1B), albeit considerably lower than the majority of chronically infected HIV-1^+^ individuals. It is debatable whether the pVL in such situations is the cause or effect of immunological changes, and although cART-treated individuals provide a control group for low pVL, and therefore lower antigenic stimulation, additional effects of cART do not enable resolution of the cause versus effect question, bringing into the equation virologic factors such as infection with less pathogenic virus and the role of viral fitness (Blankson, 2010).

In HIV-1^+^ individuals exhibiting pVL BLD in the absence of cART, in addition to robust IFN-γ producing T-cell responses, we observed strong and broad lymphoproliferative responses to a number of HIV-1 proteins and peptides, that were accompanied with robust IL-2 production and IL-2-responsiveness (Wilson et al., 2000; Imami et al., 2002). HIV-1-specific CD8 T cells also display an autocrine proliferative IL-2-producing response (Imami et al., 2002; Migueles et al., 2002, 2008). For most people treated during chronic infection, prior studies have demonstrated that there is no meaningful decay of the reservoir of HIV-1 (Siliciano et al., 2003). Initiation of cART during early stages of infection results in lower levels of viral reservoirs comparable to those observed in LTNP (Pires et al., 2004; Cellerai et al., 2011), which is not apparent if cART is started during chronic infection; strongly indicating an association between timing of cART initiation and decay of the viral reservoir. A number of therapeutic approaches are anticipated which may avoid activating latently infected CD4^+^ T cells and cells of the monocytic lineage which would increase HIV-1 replication and shedding, and potential *de novo* infection of CD4^+^ cells within host, as well as increasing risk of transmission to a new host (Richman et al., 2009; Deeks et al., 2012).

It is CD4^+^ resting T cells that harbor the greatest magnitude of hidden viral reservoirs and dictate the strategy for a very important area of future research (Siliciano and Greene, 2011; Eisele and Siliciano, 2012). This may include the use of histone deacetylase (HDAC) inhibitors such as suberoylanilide hydroxamic acid (SAHA) to increase HIV-1 gene expression as described by Archin et al. (2012). Although the study numbers were low (*n* = 8) the work described is important because it is the first demonstration that it is possible to therapeutically awaken the latent reservoir in HIV-1-infected individuals, potentially exposing infected cells to elimination by the immune system or by some other intervention. A recent report, using SAHA in an *in vitro* model system, demonstrated that stimulating the HIV-1-specific CTL prior to reactivating the latent HIV-1 appears to be a crucial step for successful eradication, which should be taken into account when aiming to achieve cure (Shan et al., 2012). In this study, prestimulation with HIV-1 Gag peptides in conjunction with IL-2 induced proliferation of HIV-1-specific CD8 T cells from patients on cART, and resulted in potent targeting of infected CD4^+^ T cells in the presence of SAHA. This highlights the potential of such approaches to reduce viral reservoirs in chronically infected HIV-1^+^ subjects.

In the context of immunotherapeutic intervention it is always important to consider the potential for restoring the full functionality of memory T cell subsets, including increased proliferative responses, and overall immune reconstitution, however such approaches should also take into account the requirement for replenishing the naïve T cell compartment (Imami et al., 2007; Herasimtschuk et al., 2008).

## Thymic Function: Maintenance and Atrophy

**Figure 2:**
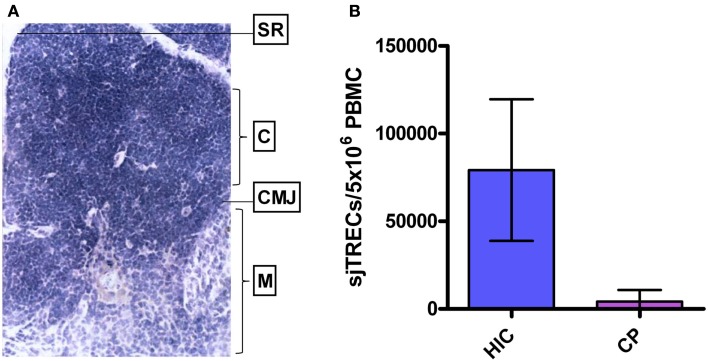
**Human thymus and thymic function in HIV-1^+^ long-term non-progressors and chronic progressors**. **(A)** Histological section of a healthy human thymus. SR, subcapsular region; C, cortex; CMJ, cortico-medullary junction; M, medulla. **(B)** TREC levels in PBMC of HIV controllers (HIC; “True LTNP”) compared to age-matched HIV-1^+^ chronic progressors (CP). Histogram plots show median values with standard deviations. Data from Imami et al. (2001), Pido-Lopez et al. (2003), and Westrop et al. (2009b).

The thymus (Figure 2A), the site where hematopoietic stem cells from the bone marrow differentiate into T cells before circulating and surveilling the periphery, is damaged by HIV-1 infection, resulting in reduced output of T cells into the periphery (Douek et al., 1998; Dion et al., 2004). HIV-1 expressing cells have been shown to be present in both the perivascular space and the true epithelial thymus of HIV-1^+^ chronically infected individuals, with evidence of thymic epithelial cell death and calcification (Haynes et al., 1999, 2000). Infection of thymic epithelial cells may result in presentation of HIV-1 epitopes as “self” to developing thymocytes, and subsequent deletion of “self-reactive” HIV-1-specific clonotypes leading to absence of these cells from the periphery. In LTNP, and other patients exhibiting stable CD4 T-cell counts throughout HIV-1 infection, the thymic epithelium, as with peripheral CD4^+^ T cells, may be resistant to HIV-1 infection (Chen et al., 2011). This offers an explanation for the observed preservation of thymic function, high output of naïve signal joint TCR excision circle-positive (sjTREC^+^) T cells, and proliferation competent HIV-1-specific T cells (Figure 2B) (Imami et al., 2001; Pido-Lopez et al., 2001; Westrop et al., 2009b). Degeneration of the thymus is characterized by replacement of the thymic tissue with adipocytes, and such age-related thymic involution has been shown to be accelerated in progressive HIV-1 infection (Douek et al., 1998; Zhang et al., 1999). Accordingly, generation of naïve CD4^+^ and CD8^+^ T cells in untreated HIV-1^+^ individuals has been shown, by sjTREC analysis, to be significantly lower in peripheral blood and lymph nodes than in age-matched uninfected controls (Douek et al., 1998; Pido-Lopez et al., 2003). This observed thymic atrophy, the high rate of T-cell turnover and the increased number of T cells in the lymphoid tissues induced by HIV-1 during chronic infection, indicates a significant role for the thymus in T-cell homeostasis during HIV-1 infection (Ho Tsong Fang et al., 2008; Bandera et al., 2010; Sasson et al., 2012).

## Virus-Specific CD8 T Cells and Proliferation

The proliferative ability of HIV-1-specific CD8^+^ T cells is associated with cytolytic capacity that is observed in LTNP only (Migueles et al., 2002). In addition to vigorous proliferation, there is a higher frequency of IL-2-producing HIV-1-specific CD8^+^ T cells in LTNP who successfully suppress viral replication (Emu et al., 2005, 2008; Pereyra et al., 2008). Polyfunctionality and defined phenotypic subsets reflect the advantage of quality, rather than quantity, of the proliferative HIV-1-specific CD8 T cells that represent mediators of immunological control (Appay et al., 2000; Gea-Banacloche et al., 2000; Betts et al., 2001, 2006; Champagne et al., 2001; Migueles et al., 2002, 2006; Addo et al., 2003). HIV-1-specific CD8 T cells in chronically infected patients are unable to mature and differentiate into fully functional proliferative and cytotoxic effector cells, due to loss of cell–cell and soluble factor mediated signals from specific anti-HIV-1 CD4 HTL and APC (Champagne et al., 2001; Kaech and Ahmed, 2003; Seder and Ahmed, 2003; Williams and Bevan, 2007). This unresponsiveness persists following initiation of cART, as HIV-1-specific CD8 T cells remain defective throughout therapy (Migueles et al., 2009). It is still not fully understood if the immune system exerts pressure on the virus to mutate away from effective immune responses, and if so, whether such mutation results in reduced viral fitness with less pathogenic potential, or results in fitter virus with the ability to escape (Gao et al., 2005; Leslie and Goulder, 2006). Thus we still question whether immune pressure on the virus may provide benefit, harm, or both to the host (Lobritz et al., 2011). Regardless, HIV-1-specific CD8 T cell proliferation relies on IL-2 production, and is consistently correlated to enhanced host immune control over viral load (Migueles et al., 2002; Zimmerli et al., 2005; Day et al., 2007). The other predictive parameter consistently associated with disease outcome has been the presence of certain MHC class I molecules; strengthening the potential role of both host genetics and MHC class I/peptide recognition by TCR on CD8 T cells (Pereyra et al., 2010; Goulder and Walker, 2012). Interestingly, while virus-specific CD8 T-cell responses in LTNP were not distinguished from those of chronic progressors on the basis of clonal diversity and/or TCR sharing as described by Mendoza et al. (2012), in another setting where viral escape from the immune response was not an issue, control of HIV-1 was associated with distinct TCR clonotypes (Chen et al., 2012). Limitations of such studies are the small numbers of patients, nevertheless these findings indicate that in addition to magnitude, it is the breadth, and also quality, affinity, and avidity of the HIV-1-specific CD8 T-cell response which are important. Additionally, certain critical epitopes restricted by protective or non-protective alleles dictate various immune responses, viral control, viral evolution, and hence diverse disease outcome (Westrop et al., 2009a; Goulder and Walker, 2012).

Evaluation of quantitative and qualitative differences accompany a number of questions about correlations between phenotype, function, and protection from disease progression. There are three main CD8^+^ T cell populations (naive, memory, and effector), which are distinguished by patterns of cell surface marker expression. Although there is no clear consensus in the use of markers to identify memory subsets of CD8^+^ T cells, several studies have used a model established by Sallusto et al. (1999), which proposes that long-lived memory CD8^+^ T cells reside within a CD3^+^CD8^+^CD45RA^−^CCR7^+^ T_CM_ cell population that is endowed with high proliferative capacity, a broad T cell repertoire, and expression of IL-7Rα (Lanzavecchia and Sallusto, 2002; Sallusto and Lanzavecchia, 2011). In contrast, effector memory CD8^+^ T cells of the T_EM_ (CD45RA^−^CCR7^−^) and T_EMRA_ (CD45RA^+^CCR7^−^) populations demonstrate strong cytolytic potential but low proliferative capacity (Sallusto et al., 1999; Lanzavecchia and Sallusto, 2002). In HIV-1 infection, patients have been shown to exhibit a skewed maturation profile of HIV-1-specific CD8^+^ T cells (Champagne et al., 2001), however the mechanisms behind this are as yet undefined (Appay et al., 2008). Upon antigenic stimulation, differentiation of naïve CD8^+^ T cells, which eventually gives rise to the different compartments of T-cell memory subsets, has a substantial effect on the CD8^+^ T cell pool (Lanzavecchia and Sallusto, 2002). Impairment of this might contribute to the dysfunctional, ineffective anti-HIV-1 response (Wherry and Ahmed, 2004), unsettling the proliferative capacity of epitope-specific CD8 T cells that are inversely related to the plasma HIV-1 RNA load (Day et al., 2007). In addition, it has been shown that removal of antigen due to either initiation of cART or development of epitope escape mutations, results in diminished HIV-1-specific CD8 T-cell response over time (Janbazian et al., 2012).

## The Interplay Between Host and Virus Genetics

Definitions of LTNP based on clinical characteristics remain arbitrary, and there is evidence for both host and non-host genetic factors as the basis of long-term non-progression. Thus it has been suggested that genomic mutations and deletions found in the host, virus, or both could account for, or contribute toward, LTNP status. Therefore, all must be taken into consideration when looking at HIV-1 infection within an individual patient. While very strong associations have been reported from a number of different cohorts between non-progressive infection/HIV-1 control and the HLA-B alleles B57 and B27 (Hendel et al., 1999; Fellay et al., 2007; Guergnon et al., 2012), not all individuals with this genotype are atypical progressors, and conversely, not all atypical progressors possess this genotype (Emu et al., 2008). Genome wide association studies (GWAS) identified SNPs within the binding groove of the MHC molecule as a high resolution explanation of the variation in viral control among patients possessing the same HLA-type (Pereyra et al., 2010). It has also been shown that the protective HLA-B*57 allele is in strong linkage disequilibrium with other genes, namely HCP5 (Fellay et al., 2007; Guergnon et al., 2012); is synergistic with particular KIR types (Martin et al., 2002); and has an effect independent to that of HLA-C (Pereyra et al., 2010).

Individuals possessing the “protective” HLA-allele B*2705 have the ability to rearrange the TCR to produce high affinity clonotypes specific to certain viral epitopes essential for HIV-1 fitness (van Bockel et al., 2011). In agreement with a number of other studies, we have observed a high representation of alleles associated with delayed disease progression in our cohort of LTNP with undetectable viral load (Guergnon et al., 2012). Separate reviews within this issue of *Frontiers in HIV and AIDS* discuss the merits of GWAS.

Cohorts of LTNP have an enrichment of MHC class I alleles associated with slow progression, and may also have a higher frequency of heterozygosity for the CCR5-Δ32 mutation, resulting in reduced HIV-1 co-receptor expression on the cell surface. However, reports on the presence of this polymorphism in LTNP cohorts and its association with HIV-1 disease progression have been varied (Poropatich and Sullivan, 2011). Attenuating mutations in the viral genes *nef* (Kirchhoff et al., 1995; Rhodes et al., 2000); *env* (Alexander et al., 2000); *gag* (Alexander et al., 2000); *rev* (Churchill et al., 2007); *vif* (Rhodes et al., 2000); *vpr* (Wang et al., 1996; Mologni et al., 2006); *vpu* (Alexander et al., 2000); and *tat* (Wang et al., 1996) have been reported. No attenuating mutations have been described in the HIV-1 reverse transcriptase (RT) enzyme, likely due to abrogation of protein function and therefore loss of virion viability if such mutations occurred. Such findings raise issues regarding viral fitness, subsequent antigenic load, and potential for mounting a fully functional proliferative T-cell response. Encouraging results from a study reporting isolation of replication-competent virus in elite controllers (normal replication kinetics in the absence of any insertions/deletions/mutations) (Blankson et al., 2007), indicate that in this instance it was the host not the virus that determined HIV-1 control (Deeks and Walker, 2007; Blankson, 2010). However, whether it is the replication competence of the virus or robustness of the T-cell responses that determine LTNP and elite controller status still remains unanswered (Lobritz et al., 2011).

## Long-Term Non-Progressors and Future Therapeutic and Prophylactic Approaches

Our current hypothesis, based on several years of experience and a large amount of preliminary data summarized herein, is that immunotherapeutic approaches designed to reverse the anergic state seen in chronic disease and induce those kinds of immune responses seen in LTNP, should improve long-term cellular functional memory leading to improved virologic control, slower disease progression, and less transmission events (Table 1). Table 1 summarizes a number of approaches, used by our group and others, with the potential to induce a LTNP-like immune profile in chronically HIV-1-infected persons; a highly desirable aim to strive for through both immunotherapy and immunization (Imami et al., 2007; Downey and Imami, 2010; Deeks et al., 2012; Shan et al., 2012). The achievement of LTNP-like status, including the reversal of both quantitative and qualitative immune defects, is likely to involve targeting of the latent viral reservoir. It is important to note that at present, the majority of these strategies are being considered in the context of fully suppressive cART, to prevent *de novo* infection of newly generated/expanded target CD4 T cells. Mimicking such efficacious natural immunity may eventually enable chronically infected individuals to stop, or at least interrupt, cART for prolonged periods.

**Table 1:** **Immunotherapeutic interventions with the potential to induce LTNP status in individuals with chronic HIV-1 infection**.

Treatment of chronic progressors	Potential immunological effects resulting from treatment aiming to induce a LTNP-like immune profile
None	Excessive immune activation and exhaustion; immunosuppression; T-cell anergy and unresponsiveness to HIV-1; infected CD4 T cells; dysfunctional APC; dysregulated NK cells
cART	Increased number of naïve T cells; functional improvement in T-cell responses to some recall antigens; partial normalization of activation, exhaustion, and regulatory function; some normalization of NK cell and APC function; incomplete reconstitution of fully functional HIV-1-specific CD4 and CD8 T-cell effector responses
Cytokines (such as IL-2)	Improved T-cell growth, survival, differentiation/maturation; reversal of T-cell anergy; increased frequency and function of T effectors and Tregs, particularly HIV-1-specific CD4 HTL and CD8 CTL; lower the numbers of HIV-1-infected latent CD4 T cells
Cytokines (such as GM-CSF)	Reversal of anergy; increased T effector cells; increased frequency of HIV-1-specific HTL and CTL; enhanced APC and NK cell function; potential to purge viral reservoirs in cells of monocytic lineage
Hormones (such as rhGH)	Increased thymic activity; increased pool of naïve T cells; decreased systemic hyperactivation; restored differentiation/maturation, prevention of apoptosis, and promotion of proliferation; increased NK cell function
HIV-1 immunogens	Provision of unpathogenic antigenic stimulation; induced/boosted anti-HIV-1 functional responses (new and memory); potential to deplete viral reservoirs (or at least reduce these to levels observed in LTNP or Elite controllers)
HDAC inhibitors	Potential to purge the latent viral reservoir in resting CD4 T cells; however stimulation of HIV-1-specific CTL prior to reactivating latent HIV-1 is thought to be crucial

*APC, antigen-presenting cell; cART, combination antiretroviral therapy; CTL, cytotoxic T lymphocyte; GM-CSF, granulocyte macrophage colony stimulating factor; HDAC, histone deacetylase; HTL, helper T lymphocyte; IL-2, interleukin-2; NK, natural killer; rhGH, recombinant human growth hormone*.

**Figure 3:**
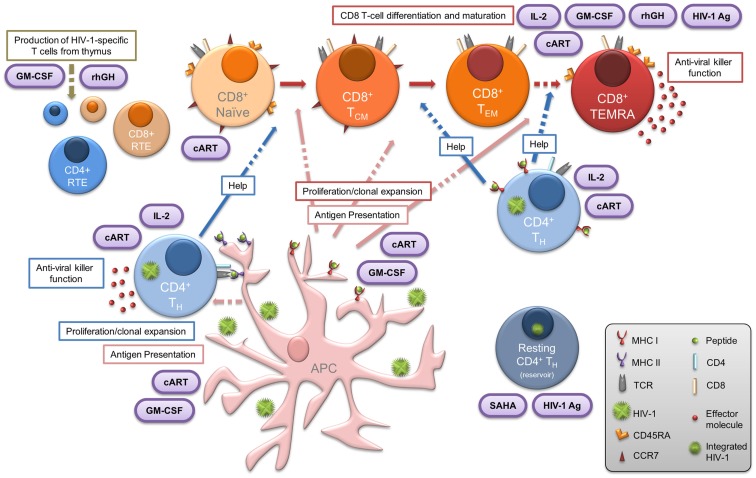
**Central importance of HIV-1-specific CD4 T cells in LTNP: summary of the various elements that contribute to, or are essential features of, the LTNP phenotype**. Dashed arrows indicate where, in chronic HIV-1 infection, the functional immune response present in LTNP is compromised. Therapeutic intervention is represented in purple ovals. Concomitant administration of immune-based therapies with effective cART may result in reversal of both the CD4 and CD8 T lymphocyte dysfunction commonly observed to persist in treated HIV-1^+^ progressors. Such immune-based therapeutic strategies in conjunction with novel approaches (including HDAC inhibitors such as SAHA) for treatment of chronic HIV-1 infection may enable the induction of virus-specific CD4 T cells essential for the subsequent “kick-start” and expansion of specific CD8 T cells. This provides a window of opportunity to steer the immune system to the advantage of the patient and achieve LTNP status or functional cure. APC, antigen-presenting cell; cART, combination antiretroviral therapy; CTL, cytotoxic T lymphocyte; GM-CSF, granulocyte macrophage colony stimulating factor; HIV-1 Ag, HIV-1 antigen; HTL, helper T lymphocyte; IL-2, interleukin-2; NK, natural killer; rhGH, recombinant human growth hormone; RTE, recent thymic emigrants; SAHA, suberoylanilide hydroxamic acid.

The primary aim of our current and future work is to determine whether the proliferative IL-2-secreting HIV-1-specific T cells in HIV-1^+^ patients exhibiting successful suppressive control over viral replication are influenced by viral fitness. We have recently described the criteria important to define cohorts internationally (Guergnon et al., 2012; Mandalia et al., 2012), along with standardized methodology for measurement of the efficacy of T-cell responses and characterization of the immune correlates of the non-progressive phenotype (Gotch et al., 2005). Further to the derivation of the salient mechanisms of suppressive anti-HIV-1 immune responses observed in LTNP, the introduction of validated assays which can be used to describe immunological phenomena in chronically infected HIV-1^+^ patients undergoing immunotherapy, are necessary to enable meaningful comparisons with responses observed in LTNP (Figure 1). We also emphasize the importance of comprehensive analyses assessing T cells directed against diverse HIV-1 proteins in order to determine the entire quantity (breadth and magnitude), and also the quality (proliferative capacity, polyfunctionality and subset phenotype) of virus-specific immune responses, enabling us to boost T-cell responses to novel epitopes. Creating gold standard LTNP status remains the ultimate aim where proliferation competent HIV-1-specific T-cell responses are induced, and maintained, with the ability to purge viral reservoirs, eradicate infection, and achieve either functional or sterilizing cure, as illustrated in Figure 3. Hence, sustaining such responses form the rationale for novel immunotherapeutic intervention in the context of cART.

It is essential that we reach consensus on several levels, including improved understanding of the kinetics of the T-cell response; the link between T-cell phenotype and proliferation; the relationship between T-cell function (IL-2 production and IL-2/IL-2R signaling) and proliferation; the association between T-cell antigen specificity and proliferation; and the effect of antigenic load on proliferation. In addition, evaluating immunoregulatory dynamics, intracellular signaling, and full complexity of the HIV-1-specific proliferative IL-2-producing response is critical. In parallel to studies concerning CD8 T cells we must also investigate the virus-specific CD4 T-cell compartment and acknowledge that the quality/quantity/affinity/avidity/breadth of the MHC class II/peptide: TCR interaction plays a role in the generation of effective balanced immunity. Furthermore, regarding the use of standardized novel methodology for assessing T-cell function, phenotype, specificity, and the immune correlates of non-progression we should consider comparing novel CFSE proliferation assays with conventional 3H-thymidine uptake assays, in addition to assessing the kinetics of IL-2 production, utilization, and consumption. In parallel, cytolytic capacity measured by the conventional chromium (51Cr) release assay can be combined with newer techniques to evaluate degranulation and cytotoxicity (namely CD107a, perforin, and granzymes). Even with this plethora of technology at our finger tips, the reliability of data will depend on the quality of the sample (fresh versus cryopreserved cells) (Gotch et al., 2005). Also, microarrays and GWAS necessitate well defined patient cohorts and good quality mRNA and DNA samples. Future development will also focus on combining flow cytometry and mass spectrometry (cytometry by time-of-flight; CyTOF) (Bandura et al., 2009; Bendall and Nolan, 2012). A recent study (Newell et al., 2012) used this method to re-examine the functional and phenotypic diversity of human CD8 T lymphocytes, and identified more than 200 functional phenotypes represented by distinct CD8 T cell subsets (Chen and Weng, 2012). It is also extremely encouraging that novel immune-monitoring models that integrate multiple functions of epitope-specific CD8 T cells, which differentiate non-progressive from progressive HIV-1 infection, list the proliferative capacity of these cells as the strongest single discriminant (Ndhlovu et al., 2013).

## Concluding Remarks

Based on novel technologies, a substantial number of parameters for antigen-specific T cells can be monitored including absolute frequencies, phenotypic subpopulations, and functional capacities such as proliferation, cytotoxicity, cytokine secretion, and degranulation. It has become increasingly clear that it is necessary to determine the parameters which represent the polyfunctionality of CD4 and CD8 T-cell responses when undertaking a meaningful immune-monitoring analysis. Immunological endpoints during immunization and immunotherapy should reflect the quality and quantity of T-cell responses definitive of LTNP status, such as those described herein. Changes in breadth, strength, and the “quality” of specific cellular responses, and reduction of viral reservoirs compared to baseline values should be included. Comparisons at all times must be drawn with similar responses in LTNP patients who naturally control disease as it is this gold standard that immunotherapy aims to induce. Evaluations of the complexity of immunological response after immunization is a challenge for investigators trying to define optimal technologies and methodologies, as well as the amplitude of their read out to define vaccine and infection-induced T-cell responses. Therefore at present, all options should be equally considered for the evaluation of potential fully functional HIV-1-specific T-cell immunological responses.

## Conflict of Interest Statement

The authors declare that the research was conducted in the absence of any commercial or financial relationships that could be construed as potential conflict of interest.

## Footnotes

^1^www.iavi.org

^2^www.hvtn.org
